# *Clostridioides difficile* Among Asymptomatic Onco-Hematological Carriers: Risk Factors, Molecular Characterization, and Antibiotic Resistance

**DOI:** 10.3390/antibiotics15090939

**Published:** 2026-09-21

**Authors:** Ana Martín Bermúdez, Sara Milosevic, Maria Jose Ramos-Real, Eduardo Salido, Jose Luis Irribarren, Maria Lecuona, Ana López-Lirola, Enrique González Dávila, Bernardo Gonzalez, Rosa Garcia, Miriam Hernández-Porto

**Affiliations:** 1 Department of Microbiology, Hospital Universitario de Canarias, 38320 San Cristobal de La Laguna, Tenerife, Spain; mjoseramosreal@hotmail.com (M.J.R.-R.); mlecuona2005@yahoo.es (M.L.); mhernapo@ull.edu.es (M.H.-P.); 2 Center for Rare Diseases (CIBERER), Universidad de La Laguna, 38320 San Cristobal de La Laguna, Tenerife, Spain; saramilosevic.08@gmail.com; 3 Department of Pathology, Hospital Universitario de Canarias, 38320 San Cristobal de La Laguna, Tenerife, Spain; edsalido@gmail.com; 4 Department of Critical Care, Hospital Universitario de Canarias, 38320 San Cristobal de La Laguna, Tenerife, Spain; joseluis.iribarren@gmail.com; 5 Infectious Diseases Department, Hospital Universitario de Canarias, 38320 San Cristobal de La Laguna, Tenerife, Spain; lopezlirola@hotmail.com; 6 Mathematics, Statistics and Operations Research Department, IMAULL Institute, Universidad de La Laguna, 38206 San Cristobal de La Laguna, Tenerife, Spain; egonzale@ull.edu.es; 7 Hematology Department, Hospital Universitario de Canarias, 38320 San Cristobal de La Laguna, Tenerife, Spain; bjgonzalez@telefonica.net; 8 Oncology Department, Hospital Universitario de Canarias, 38320 San Cristobal de La Laguna, Tenerife, Spain; rdgmarrero@gmail.com; 9 Department of Obstetrics, Gynecology, Pediatrics, Preventive Medicine and Public Health, Facultad de Ciencias de la Salud, Universidad de La Laguna, 38200 San Cristobal de La Laguna, Tenerife, Spain

**Keywords:** *Clostridioides difficile*, asymptomatic colonization, onco-hematological patients, whole-genome sequencing

## Abstract

**Background/Objectives:** *Clostridioides difficile* colonization is common among hospitalized onco-hematological patients, yet its clinical significance and risk factors in this population remain incompletely characterized. This study aimed to determine the prevalence, risk factors, and genomic characteristics of *C. difficile* colonization among oncology and hematology inpatients, and to assess its relationship with progression to symptomatic infection. **Methods:** We conducted a prospective cohort study of patients admitted to the oncology and hematology departments of a tertiary-care hospital in the Canary Islands, Spain, between March and October 2025. Weekly active screening for *C. difficile* colonization was performed on stool samples throughout hospitalization. Risk factors were assessed using multivariable logistic regression, and whole-genome sequencing was performed on 16 of the 23 recovered isolates (after excluding 3 non-recoverable and 4 contaminated isolates) to characterize sequence types, ribotypes, and antimicrobial resistance determinants. **Results:** Among 215 patients (262 admission episodes), colonization was detected in 23 episodes (8.8%), with higher rates in oncology (10.9%) than hematology (7.2%; adjusted OR 0.37, 95% CI 0.14–0.96, *p* = 0.042). Chemotherapy administered during hospitalization was the strongest independent risk factor for colonization (adjusted OR 4.16, 95% CI 1.56–11.10, *p* = 0.004). Only 4.3% of colonizations were classified as truly community-associated, with 95.7% of patients showing documented healthcare contact. Sequencing of 16 isolates identified nine sequence types, including ST11 (ribotype 078, of zoonotic origin) and rare ribotypes (RT010, RT451); four isolates (36%) exhibited moxifloxacin resistance linked to *gyrA*/*gyrB* mutations, while all remained susceptible to vancomycin and metronidazole. No colonized patient progressed to symptomatic infection; the single CDI case in the cohort occurred in a non-colonized patient. **Conclusions:** These findings support current recommendations against isolating or treating asymptomatically colonized patients, while underscoring the need for continued genomic surveillance and reinforced environmental hygiene and antimicrobial stewardship in this high-risk population.

## 1. Introduction

*Clostridioides difficile* is a Gram-positive, anaerobic, spore-forming bacillus and the leading cause of healthcare-associated infectious diarrhea worldwide, capable of producing severe complications such as pseudomembranous colitis, toxic megacolon, and sepsis in vulnerable populations [1].

*C. difficile* infections (CDI) have a strong impact on the EU/EEA population. The European Centre for Disease Prevention and Control estimates that in 2016 and 2017 there were roughly 189,526 cases of healthcare-associated *C. difficile* infection (HA CDI) in acute care hospitals annually, with an estimated 7419 fatal HA CDI cases per year, with CDI as a possible contributing factor [2,3].

Its clinical importance is underscored by its ability to cause conditions ranging from healthcare-associated diarrhea to pseudomembranous colitis and fulminant colitis, which occurs in up to 5% of cases [4]. Its association with antimicrobial consumption, propensity for transmission in healthcare settings, the inefficiency of conventional cleaning methods using detergents to control its spread, and elevated recurrence rates in higher-risk populations necessitate close surveillance of this pathogen in clinical facilities [5,6,7,8]. The development of CDI appears to depend on a multitude of factors, including prior colonization by *C. difficile*; however, this remains somewhat controversial, as some authors argue that this risk depends on whether the colonization is caused by a toxigenic strain or not [9].

Resistance in *C. difficile* to therapeutic agents such as vancomycin and metronidazole remains rare, though not negligible, with reported rates generally below 5%, arising through mechanisms including acquisition of resistance genes. In contrast, resistance to non-therapeutic antibiotics such as fluoroquinolones is considerably more prevalent and epidemiologically relevant, given their role as a major risk factor for CDI development and their association with the emergence and spread of hypervirulent strains. [10].

Indeed, authors such as Natarajan et al. described a protective role of colonization against the development of subsequent CDI in patients colonized by non-toxigenic *C. difficile* strains [11].

This role could be explained by the production of antibodies against *C. difficile* cell-wall-surface proteins, both in colonized patients and in patients who have recovered from a CDI; therefore, this protection may not be exclusive to non-toxigenic strains [11,12,13].

*C. difficile* colonization rates vary depending on the region studied as well as the target population. Thus, colonization rates in healthy adults, patients upon hospital admission, hospitalized patients, residents in long-term care facilities, and healthcare workers are estimated at 4–15%, 3–21%, 3–21%, 4–51%, and 0–13%, respectively [14]. In hemato-oncological populations specifically, colonization rates at hospital admission have been reported to range from 9.3% to 14%, with up to 13.3% of colonized patients progressing to symptomatic infection during hospitalization [15].

Therefore, the present study aimed to investigate, in the onco-hematological population of our institution, *C. difficile* colonization rates, risk factors for colonization, the significance of prior colonization by toxigenic versus non-toxigenic strains for the subsequent development of CDI, and the molecular characterization and antibiotic resistance of our isolates.

## 2. Results

### 2.1. Colonization C. difficile; Risk Factors

A total of 215 onco-hematological patients were enrolled in the study, accounting for 262 hospital admission episodes across the oncology and hematology departments (Table 1). A total of 23 episodes (8.8%, involving 23 individual patients) demonstrated *C. difficile* colonization, and NTCD (non-toxigenic *C. difficile*) was present in six (26.1%) episodes (Table 2). When stratified by service, the colonization rate was 12/110 (10.9%) among oncology episodes and 11/152 (7.2%) among hematology episodes. Four colonized patients had more than one hospital admission episode; however, colonization was always detected during the last recorded episode.

Of the 20 candidate predictors screened individually (Table S1), only hospital service and chemotherapy administration during hospitalization remained independently associated with *C. difficile* colonization in the multivariable generalized linear mixed model (Table 3). Given the limited number of colonization events (23 of 262 episodes), the multivariable model was restricted to these two predictors to avoid overfitting.

Colonization was first detected at a median of 5 days after admission (range: 0 to 130 days). Among the 23 colonization episodes, 1/23 colonizations (4.3%) were community-associated (CA), 7/23 (30.4%) were community-onset but healthcare-facility-associated (CO-HCFA), and 15/23 (65.2%) were healthcare-facility-onset (HCFO), of which 8/23 (34.8%) were early (72 h–14 days) and 7/23 (30.4%) were late (≥14 days after admission). Of the 23 patients colonized with *C. difficile* (CD), none developed CDI during follow-up. In contrast, one patient who was not colonized at admission developed CDI during hospitalization.

All-cause 90-day mortality did not differ significantly between colonized and non-colonized patients (7/23, 30.4% vs. 61/192, 31.8%; Fisher’s exact test, *p* = 1.000; OR 0.94, 95% CI 0.31–2.57).

During the study period, only one episode (0.4% of 262 episodes) progressed to clinically confirmed CDI. This case occurred in a non-colonized hematology patient, who was a 56-year-old woman with underlying cardiovascular disease and obesity, with documented prior hospitalization and non-hospital healthcare exposure in the six months preceding admission, and who was receiving proton pump inhibitors and antibiotic therapy during the index admission. No colonized patient (0/23) progressed to clinical infection. Fisher’s exact test showed no significant association between colonization status and progression to CDI (*p* = 1.000); the odds ratio could not be reliably estimated due to the zero-cell count in the colonized group.

### 2.2. Molecular Characterization and Acquired Resistance and Virulence Genes

**Figure 1:**
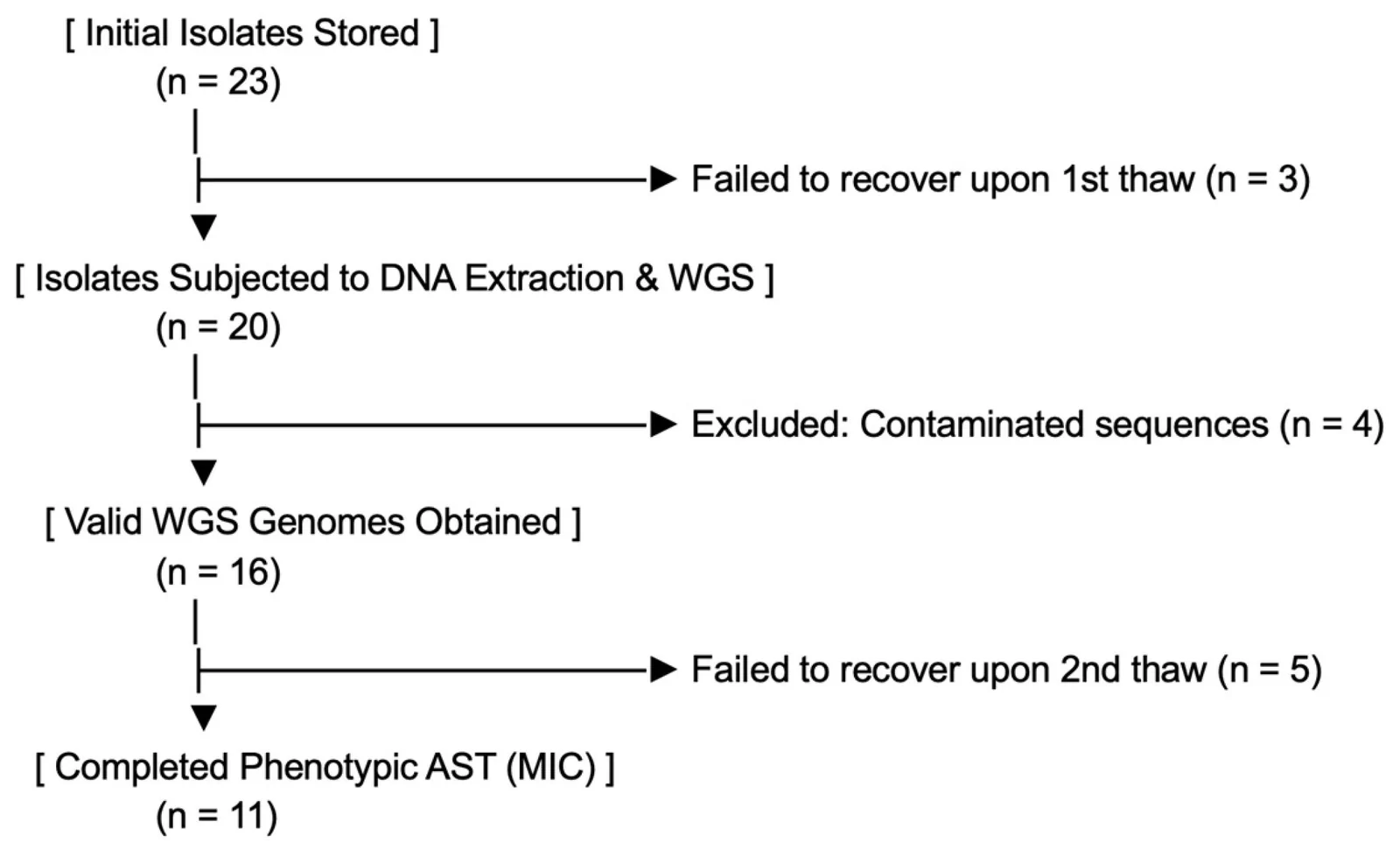
Flowchart of *Clostridioides difficile* isolate selection for whole-genome sequencing and antimicrobial susceptibility testing.

Of 23 *C. difficile* isolates initially collected, three could not be recovered from frozen storage and were excluded prior to sequencing. Whole-genome sequencing was performed on the remaining 20 isolates. Following sequencing, four isolates were identified as contaminated with non-*C. difficile* DNA (based on bimodal per-contig coverage and GC-content distributions) and were excluded from downstream analysis, yielding a final set of 16 isolates. Of these, 11 isolates were successfully subcultured and subjected to phenotypic antimicrobial susceptibility testing (MIC determination); the remaining five isolates failed to grow on subculture and were therefore excluded from phenotypic testing (Figure 1).

The most prevalent sequence types (STs) were ST2 and ST42 (18.7%, 3/16 each), followed by ST11, ST238, and ST15 (12.5%, 2/16), and ST55, ST12, ST458, and ST26 (6.25%, 1/16). The distribution of MLSTs, ribotypes, and gene toxin profiles is shown in Table 4.

### 2.3. Phylogenetic Analyses

**Figure 2:**
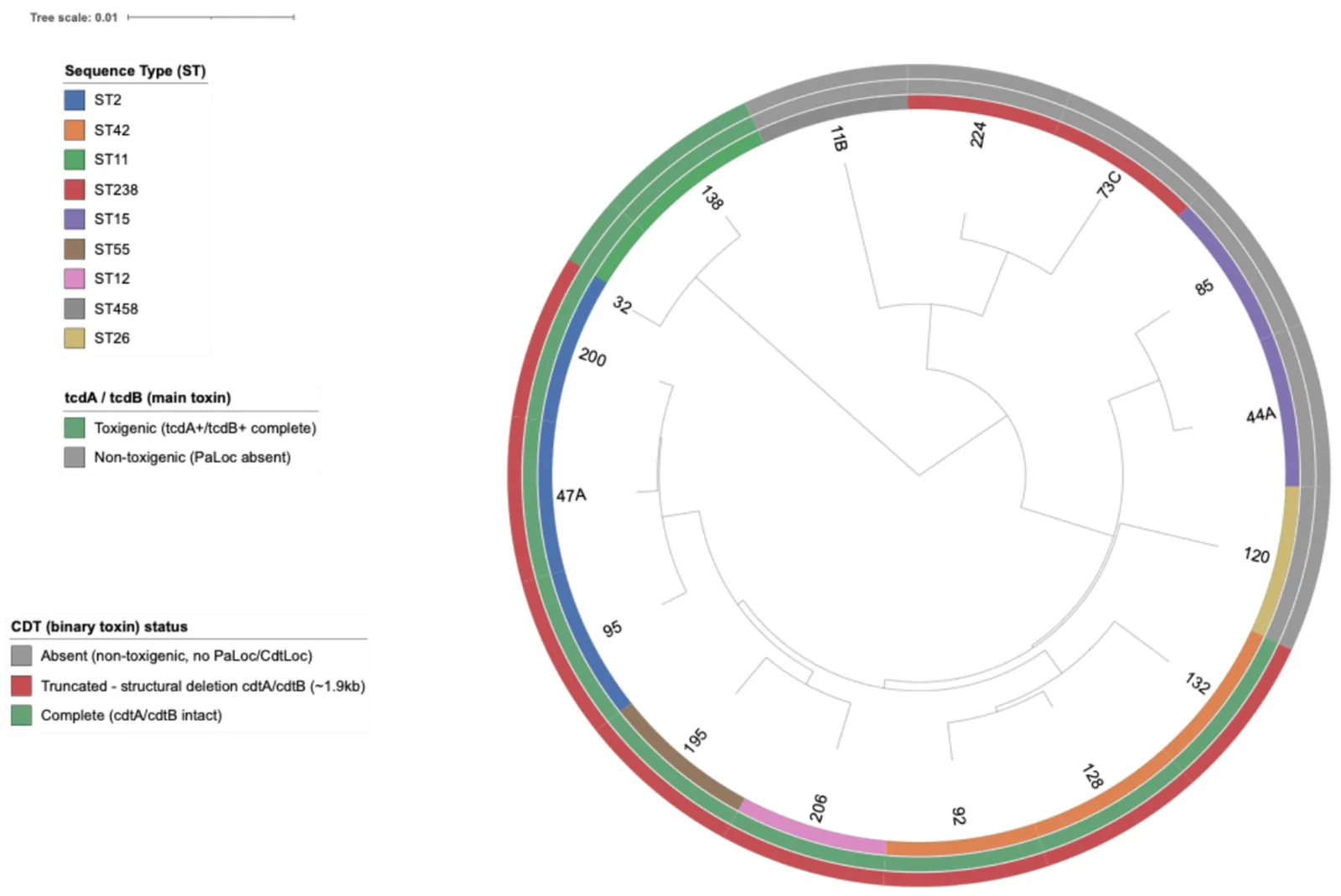
The phylogenetic tree of *C. difficile* strains isolated from onco-hematological patients.

Phylogenomic analysis of the 16 strains grouped them into the established *C. difficile* clades (Figure 2): Clade 5 (ST11), Clade 4 (ST238, ST458), and Clade 1 (ST2, ST12, ST15, ST26, ST42, ST55).

**Figure 3:**
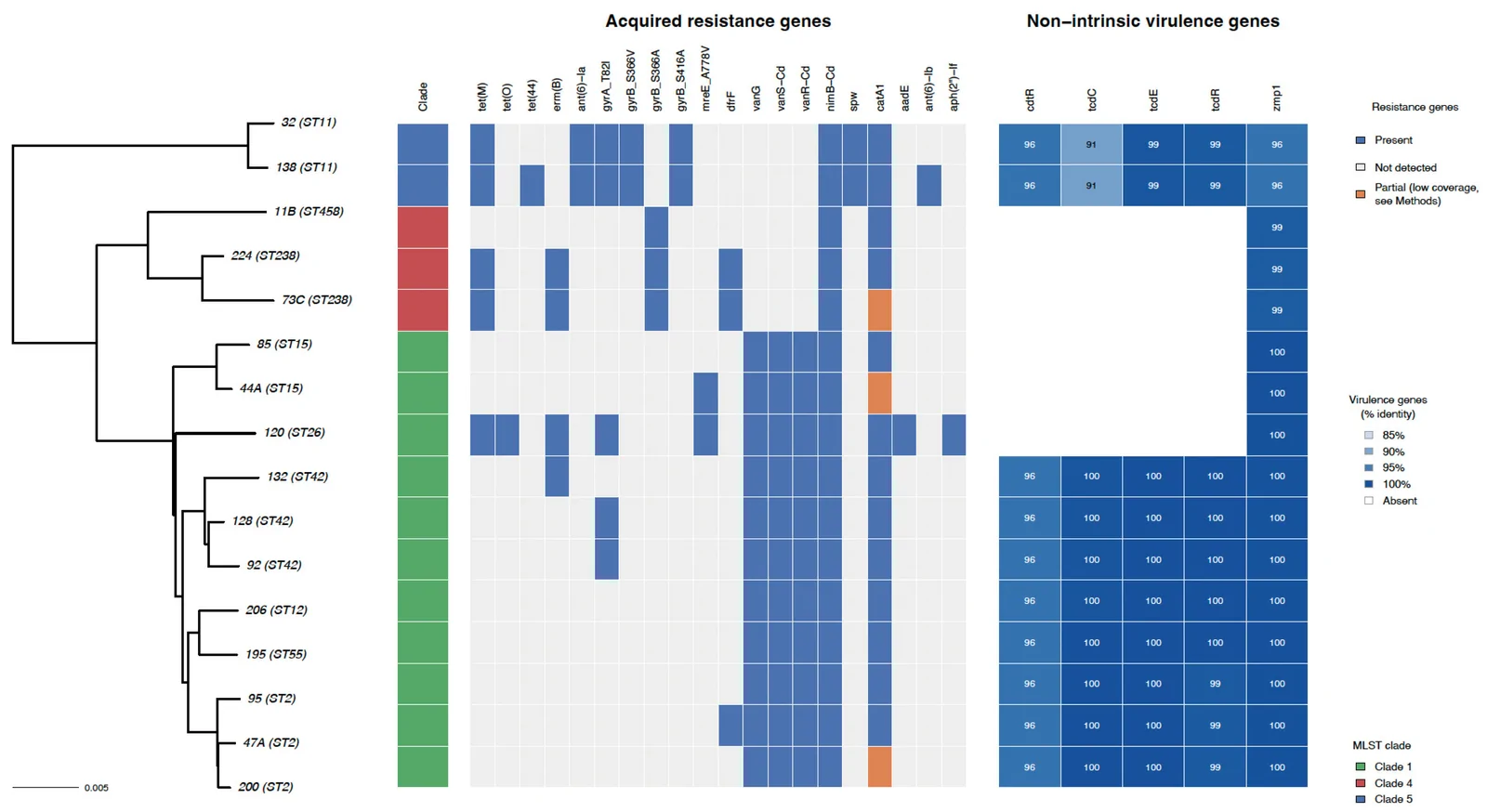
Neighbor-joining phylogenetic tree, based on genome-wide k-mer distance, showing the phylogenetic relationships among the *C. difficile* isolates alongside their acquired resistance gene and non-intrinsic virulence gene content. Tip labels indicate the isolate and its sequence type (ST), and the colored column indicates the MLST clade. Numbers in the virulence gene heatmap indicate percent identity. The scale bar represents a genome-wide k-mer distance of 0.005. Partial catA1 detections (orange) correspond to low coverage (<3× compared with 50–250× genome-wide) and to hits at contig boundaries; genomic completeness was not confirmed.

We identified five non-intrinsic virulence genes displaying diverse functional roles (Figure 3). These included regulatory genes involved in controlling toxin expression (*tcdC*, *cdtR*, and *tcdR*), a gene responsible for toxin secretion (*tcdE)*, and a factor associated with host colonization and immune evasion (*zmp1*). The *zmp1* gene was harbored by all strains, while toxin-regulatory genes were restricted to all toxigenic isolates.

### 2.4. Antimicrobial Resistance

**Figure 4:**
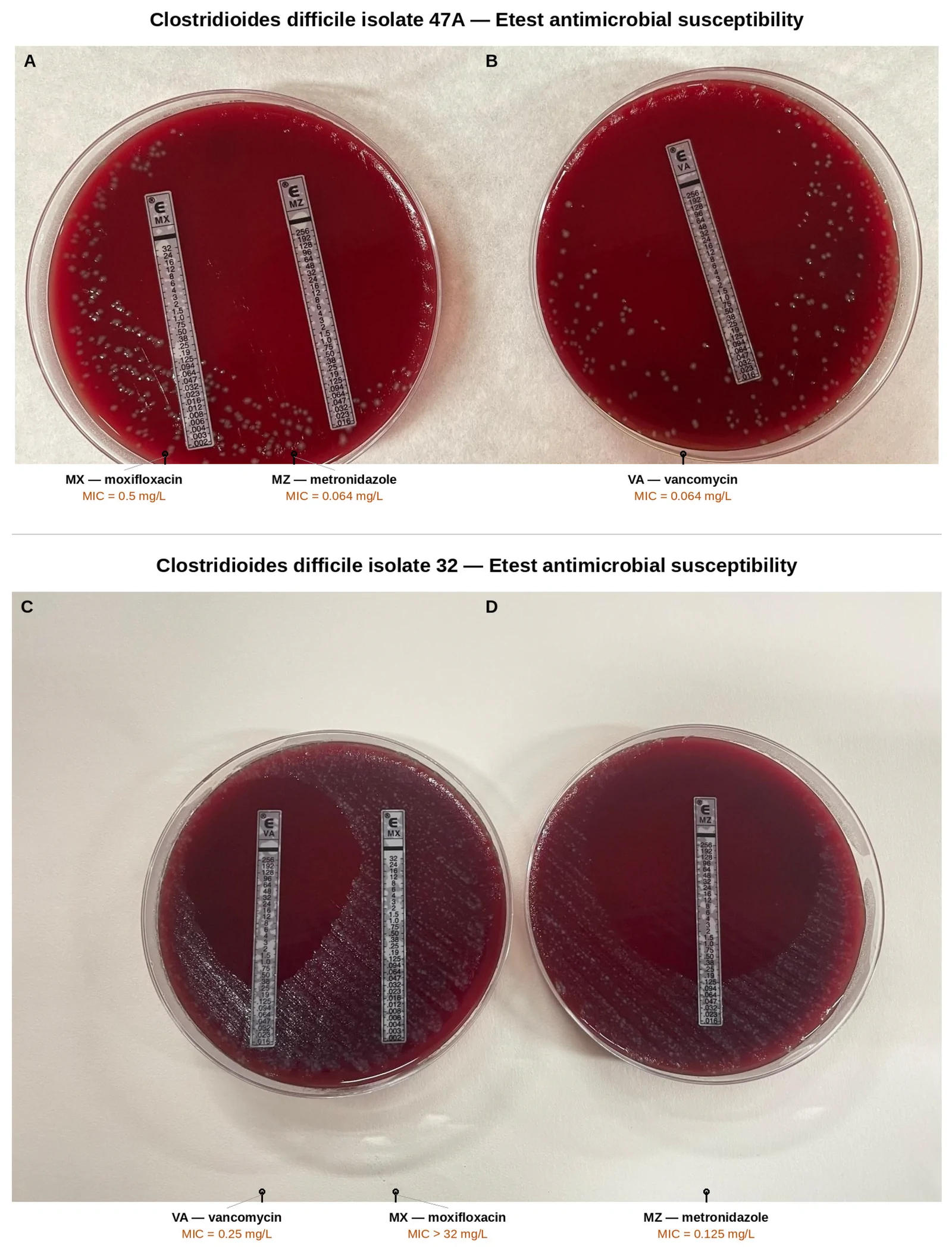
Susceptibility phenotypes to vancomycin, metronidazole, and moxifloxacin in *C. difficile* strains determined by Etest. (**A**) Isolate 47A, moxifloxacin (MX; MIC 0.5 mg/L) and metronidazole (MZ; MIC 0.064 mg/L); (**B**) isolate 47A, vancomycin (VA; MIC 0.064 mg/L); (**C**) isolate 32, vancomycin (VA; MIC 0.25 mg/L) and moxifloxacin (MX; MIC > 32 mg/L); (**D**) isolate 32, metronidazole (MZ; MIC 0.125 mg/L).

The MICs of different antimicrobial agents against *C. difficile* isolates are presented in Table 5. It was noted that MIC determination was not possible for five isolates because the strains could not be recovered at the time of phenotypic susceptibility testing. All isolates were sensitive to metronidazole and vancomycin, but four (36%) exceeded the moxifloxacin ECOFF value (Figure 4).

It was noted that none of the isolates displaying high moxifloxacin MICs were associated with fluoroquinolone exposure within two months prior to colonization. Nevertheless, only one patient had received fluoroquinolone treatment during their hospital admission. Furthermore, none of the patients co-occurred in time or space during hospitalization.

Among the 20 acquired/point-mutation resistance genes screened, *nimB-Cd* and *catA1* were the most prevalent (16/16, 100%), followed by *vanG*, *vanS-Cd* and *vanR-Cd* (11/16, 68.8% each); *tet(M)* and *gyrA*_T82I (5/16, 31.2% each); *erm(B)* (4/16, 25.0%); *gyrB*_S366A and *dfrF* (3/16, 18.8% each); *ant(6)-Ia*, *gyrB*_S366V, *gyrB*_S416A, *mreE*_A778V and *spw* (2/16, 12.5% each); and finally, *tet(O)*, *tet(44)*, *aadE*, *ant(6)-Ib* and *aph(2*″*)-If*, each detected in a single isolate (1/16, 6.2%).

The four isolates exhibiting moxifloxacin MICs above the ECOFV value (strains 32, 92, 128, and 224) all carried mutations in gyrA and/or gyrB (gyrA_T82I and/or gyrB_S366A/S366V/S416A), consistent with the established genetic basis of fluoroquinolone resistance in *C. difficile* [10]. Notably, gyrA_T82I was detected in 5/16 isolates (31.2%), one more than the number of phenotypically resistant isolates, as MIC determination was not possible for that isolate (strain 138) due to failure to recover the strain for phenotypic testing. Strains carrying the isolated GyrB S366A substitution, in the absence of concomitant gyrA mutations, displayed a predominantly susceptible or low-level resistance phenotype, except for strain 224 (ST238), which exhibited a moxifloxacin MIC of 12 mg/L—approximately 50-fold higher than that of strain 73C, which carries the same isolated substitution.

Despite the high prevalence of vanG, vanS-Cd and vanR-Cd (11/16, 68.8%) and of nimB-Cd (16/16, 100%), all isolates remained phenotypically susceptible to vancomycin and metronidazole. No promoter mutations associated with constitutive nimB expression were detected among our isolates.

## 3. Discussion

### 3.1. Colonization Rates in Oncology and Hematology Patients

The overall *C. difficile* colonization rate observed in our cohort (23/262 episodes, 8.8%) falls within the range previously reported in hospitalized onco-hematological patients. When stratified by service, colonization was numerically higher in oncology (12/110, 10.9%) than in hematology (11/152, 7.2%), a difference that reached statistical significance in the adjusted model (aOR 0.37, 95% CI 0.14–0.96, *p* = 0.042). Our figures are consistent with those reported by Cannon et al., who found a 9.3% prevalence of toxigenic *C. difficile* colonization among hematology/BMT patients at admission and with de-La-Rosa-Martínez et al., who described rates of 9–16% in a cancer population [19,20]. A recent systematic review and meta-analysis including 51 studies estimated a pooled prevalence of asymptomatic toxigenic *C. difficile* carriage of 12.1% (95% CI, 10.5–13.9%) among patients with cancer, further supporting the plausibility of our findings [21].

### 3.2. Risk Factors for C. difficile Colonization

Chemotherapy administered during hospitalization was the strongest independent predictor of colonization (aOR 4.16, 95% CI 1.56–11.10, *p* = 0.004), an effect driven mainly by oncology patients (58.3% vs. 17.3% chemotherapy exposure among colonized vs. non-colonized, *p* = 0.004 in the oncology-stratified subgroup, Table 2), consistent with reports linking cytotoxic and immunosuppressive therapy to disruption of colonization resistance in cancer patients [22,23]. Admission to the oncology service was likewise independently associated with higher odds of *C. difficile* colonization than admission to hematology (hematology vs. oncology: aOR 0.37, 95% CI 0.14–0.96; *p* = 0.042); notably, this association was not present in univariable analysis (OR 0.64, 95% CI 0.27–1.50, *p* = 0.303) and emerged only after adjusting for chemotherapy administration. This finding differs from most published studies comparing these two populations, which, when differences have been observed, have generally reported a higher risk among patients with hematological malignancies than among those with solid tumors, whereas other studies have found no significant differences [14]. However, most previous research has focused on risk factors for symptomatic *C. difficile* infection rather than asymptomatic colonization, and the determinants of these two outcomes may not be identical. Overall, the identification of chemotherapy as the strongest predictor of colonization was an expected finding, consistent with its established role in disrupting colonization resistance. In contrast, the emergence of hospital service as a significant predictor only after adjustment for chemotherapy—but not in the univariable analysis—was a comparatively less anticipated finding, highlighting the confounding effect of chemotherapy exposure on this association.

Two well-established risk factors were not significantly associated with colonization in our cohort, most likely because of the limited variability in exposure rather than a true absence of association. Both proton pump inhibitor use and antibiotic exposure are among the most consistently reported predictors of *C. difficile* colonization, yet neither discriminated colonized from non-colonized patients in our study, as both were highly prevalent across all groups (PPIs: 91.7–100%; antibiotics: 64.3–90.9%), leaving little exposure contrast to detect an association [24].

### 3.3. Timing and Origin of Colonization

Beyond individual risk factors, the timing and origin of colonization further illustrate the pervasive influence of the healthcare setting in this population. Only 1/23 colonizations (4.3%) were classified as truly community-associated, with the remaining 22 patients (95.7%) showing some documented healthcare contact—either during the current admission or in the preceding 6 months—underscoring the pervasive role of the healthcare system as a reservoir for *C. difficile* acquisition in this population, consistent with recent evidence that the majority of apparently community-associated CDI cases have documented recent healthcare contact [25,26]. The predominance of healthcare-associated colonization was expected, given the increasing recognition that most apparently community-onset CDI cases have documented recent healthcare contact. However, the near-equal distribution between early and late acquisition was somewhat unexpected, as it did not reveal a single dominant temporal pattern. Among colonizations detected during the current admission, timing was distributed similarly between early (8/23, 34.8%; 72 h–14 days) and late acquisition (7/23, 30.4%; ≥14 days), in line with the early/late distinction described by McFarland et al. for nosocomial *C. difficile* acquisition [27]. Taken together, these findings suggest that colonization risk was not confined to a discrete point-source exposure early after admission, but rather reflected continuous, cumulative contact with the healthcare environment throughout—and even before—hospitalization.

### 3.4. Molecular Characterization

Having characterized the epidemiological patterns of colonization, we next examined the molecular features of the colonizing isolates among colonization cases that occurred from March to October 2025 in the hematology and oncology wards of a tertiary-care hospital in the Canary Islands, Spain. A limited number of MLST groups were identified—ST2 (18,75%), ST42 (18,75%), ST11 (12,5%), ST238 (12,5%), ST15 (12,5%), ST55 (6.25%), ST12 (6,25%), ST458 (6,25%), and ST 26 (6.25%)—with the majority of colonizing strains being derived from clade 1, the most prevalent and genetically diverse clade in human *C. difficile* infections. This diversity, with no dominant clone identified, suggests that colonization in this cohort resulted from multiple independent acquisition events rather than transmission from a single clonal source. However, this observation does not rule out the possibility of a broader environmental reservoir within the hospital contributing to strain diversity, as *C. difficile* spores can persist for extended periods on surfaces and in the healthcare environment. Dedicated environmental surveillance studies actively searching for and characterizing environmental reservoirs would be needed to clarify their potential role in sustaining this diversity [28,29]. PCR ribotyping revealed a degree of diversity in *C. difficile* strains commonly found in European settings, except for RT010 and RT451. RT010 is probably little known due to being a non-toxigenic ribotype, and RT451 is a rare toxigenic ribotype seldom reported in European hospital surveillance. Predominant PCR ribotypes included those typically associated with transmission among patients in healthcare facilities (ribotypes 106, 020) and highlights their enduring prevalence and adaptability [30,31].

Likewise, we identified ribotypes with potential for zoonotic transfer, such as RT 003 and 078. ST11 (RT078) is often described as a hypervirulent, community-acquired strain of zoonotic or porcine origin [32]. Toxigenic phenotypes were consistent within MLST categories, in agreement with prior reports, except for the presence of binary toxin in ribotype 003 [16,31,33]. The ribotypes 020, 106, 451, and 106 were characterized by carrying a truncated 2.3 kb fragment in the CDT locus; this is a non-functional truncated form of the binary toxin (CDT) locus [34].

Notably, strains 138 and 32 (ST11) exhibited the *tcdC*-Δ18 genotype, which is characterized by a nonsense point mutation at nucleotide position 184 (*C184T*) alongside a 39 bp deletion (positions 341–379). These findings support the possibility that the marked alterations observed in TcdC lead to impaired protein function in these strains, thereby contributing to increased toxin expression [35]. Our sequencing analysis revealed that the *cdtR* gene in these strains contains a naturally occurring stop codon mutation at codon 322, which is likely to result in a non-functional protein [36].

Virulence gene *zmp1* was prevalent in 100% of isolates, showing the essential role of Zmp1 (extracellular metalloprotease) in the adhesion of *C. difficile* to human cell-surface proteins [37].

More broadly, resistance to a broad range of antibiotics allows *C. difficile* to colonize and infect the host in the presence of antimicrobials. Rates of antimicrobial resistance in *C. difficile* vary in different geographic regions [38].

In our study, we found that all phenotypically tested isolates were susceptible to metronidazole and vancomycin, despite all of them harboring the *nimB* gene and those belonging to Clade 1 harboring the *vanG* gene cluster. However, four isolates exhibited phenotypes resistant to moxifloxacin, with MIC > 4 mg/L. Several antimicrobial resistance patterns were observed according to the analyzed *C. difficile* clade. Strains from Clade 1 included ST2, ST12, ST15, ST26, ST42, and ST55. These isolates carry a functional vancomycin-resistance *vanG* gene cluster (designated as *vanGCd*), which is closely related to the *vanG* operon in *Enterococcus faecalis.* Indeed, about 85% of *C. difficile* carry this *vanG* gene cluster [39]. This clade is the main one responsible for the disease burden in humans in regions with high medical surveillance (such as North America and Europe) and is most frequently found in environments where treatments are administered, which could explain the acquisition of the *vanG* gene cluster [40]. However, vancomycin-induced expression of *vanGCd* does not confer resistance in *C. difficile*, whereas mutations in *C. difficile* VanS/RCd promote constitutive expression of *vanGCd* in vancomycin-resistant clinical isolates of ribotype 027 [41].

The strains from Clade 5, ST11, have been identified as a significant lineage associated with zoonotic transmission, with a notable prevalence in human and animal populations. These isolates are characterized by exhibiting resistance to tetracyclines and fluorquinolones [42].

In our study, we detected the presence of the point mutations *gyrA_T82I*, *gyrB_S366V* and *gyrB_S416A*. The resistance to moxifloxacin appears to be specifically due to the *gyrA_T82I* mutation [43]. This represents a significant public health concern because these resistance traits can facilitate the survival of these strains in the presence of commonly used antibiotics. Notably, strain 224 (ST238) exhibited a markedly elevated moxifloxacin MIC (12 mg/L) compared to strain 73C, despite both isolates carrying the same isolated GyrB S366A substitution. This finding illustrates that the phenotypic effect of this substitution was not entirely consistent across isolates in our cohort.

In addition, the mere presence of the *nimB* gene does not imply resistance on its own; for metronidazole resistance to manifest, a specific mutation described by Olaitan et al. in its promoter is required, which was not detected in our isolates [44].

Among the Clade 4 strains, we identified ST238 and ST458. Both sequence types are uncommon, neither was found to be toxigenic, and they exhibited discordant resistance gene profiles.

### 3.5. Colonization Status and Risk of Progression to CDI

Beyond strain characterization, a central clinical question is whether colonization itself predicts progression to disease. No association between colonization and subsequent CDI was observed in our cohort; however, only one CDI event occurred during follow-up, precluding a meaningful assessment of this relationship. Consequently, the absence of a statistically significant association should be interpreted as reflecting limited statistical power rather than evidence against an association between colonization and subsequent infection.

Although previous studies have described colonization with toxigenic strains as a risk factor for subsequent CDI, larger prospective cohorts have shown that only a minority of colonized patients subsequently develop CDI (13.4% in one recent study), indicating that progression from colonization to disease is uncommon and influenced by factors beyond colonization status alone [45,46].

Several mechanisms have been proposed to explain why only a subset of colonized individuals progress to CDI. Colonization with non-toxigenic *C. difficile* has been shown to competitively exclude toxigenic strains and reduce the risk of CDI in both experimental and clinical studies [13,47,48,49]. These mechanisms may contribute to the heterogeneous clinical outcomes observed after colonization, although they were not evaluated in the present study.

There is some controversy regarding whether *C. difficile*-colonized individuals have an increased risk of developing subsequent CDI, or if they are protected against the disease. On one hand, some authors consider that patients colonized at admission to a hospital are a considerable reservoir for *C. difficile* and, importantly, a potential source of nosocomial transmission [9,25]. On the other hand, current guidelines—including those from the IDSA and ESCMID—do not recommend isolation or decolonization treatment for colonized patients [50,51].

In fact, authors such as Guerrero et al. demonstrated that rectal and skin swabs from hospitalized, colonized patients yielded much lower counts than those from subjects with diarrhea, suggesting reduced transmission potential associated with colonized individuals. Furthermore, the colonization state seems to resolve spontaneously over time [52].

Likewise, various factors can influence the development of CDI, such as colonization by a toxigenic versus a non-toxigenic strain, the timing of colonization (during hospitalization or upon admission), and colonization by hypervirulent ribotypes [14].

Authors such as Natarajan et al. described a protective role of colonization against the development of subsequent CDI in patients colonized by non-toxigenic *C. difficile* strains; however, recent studies show that antibodies to TcdA and TcdB do not protect from colonization, but they influence disease susceptibility and, subsequently, the progression from colonization to CDI [13,52].

Therefore, acquired immunity (e.g., due to previous hospitalizations) confers resistance to the development of symptomatic CDI [14].

### 3.6. Strengths and Clinical Implications

This study offers several contributions to the understanding of *C. difficile* colonization in onco-hematological patients. First, by combining prospective clinical surveillance with whole-genome sequencing, we provide an integrated epidemiological and molecular characterization of colonizing isolates in a population at particularly high risk of CDI, an approach seldom applied in this specific clinical setting. Second, our findings highlight that healthcare contact—rather than community exposure—accounts for the vast majority of colonization events, reinforcing the role of the hospital environment as a reservoir and supporting the prioritization of environmental infection control measures in oncology and hematology wards. Third, the low observed rate of progression from colonization to symptomatic CDI contributes real-world evidence relevant to ongoing discussions regarding the clinical management of asymptomatically colonized patients, supporting current guideline recommendations against routine isolation or decolonization.

Beyond these direct implications, our genomic characterization of *gyrA*/*gyrB*-resistance mutations also opens a promising avenue for future research: tracking the prevalence of these mutations over time could, in principle, serve as a genotype-based indicator to monitor the impact of antimicrobial stewardship programs on fluoroquinolone use, an approach not explored in the present study but worth pursuing in future surveillance efforts.

### 3.7. Limitations

The interpretation of these findings should take into account several study limitations. First, fecal toxin load was not quantified, precluding assessment of its relationship with clinical outcomes. Second, the small number of colonized patients (n = 23) and the occurrence of only one CDI event during follow-up substantially limited the statistical power of our risk-factor and outcome analyses; in particular, the study lacks the statistical power necessary to conclude an absence of association between asymptomatic colonization at admission and subsequent risk of CDI (type II error risk). Third, full molecular and phenotypic characterization was not possible for all isolates, which may have introduced some selection bias in the strain-level findings. Fourth, the mechanisms proposed to underlie progression from colonization to CDI—including competition by non-toxigenic strains and host antitoxin antibody responses—were not directly assessed in our cohort. Finally, this was a single-center study conducted over a limited period, which may affect the generalizability of our findings to other institutions or patient populations.

## 4. Materials and Methods

### 4.1. Study Design and Population

This was a cohort prospective study on *C. difficile* colonization in patients admitted to the oncology or hematology departments between March and October 2025, conducted at the Hospital Universitario de Canarias, Tenerife, Canary Islands, Spain. The exclusion criteria included: (i) patients with documented CDI within the first 72 h of admission, (ii) patients with a length of stay exceeding one week prior to the first screening, and (iii) pediatric patients. Patient characteristics and examination results were extracted from medical records and included the following: demographics (age, sex, etc.), clinical parameters (underlying onco-hematological disease), comorbidities (diabetes, cardiovascular disease, obesity, hypothyroidism, dyslipidemia, chronic lung disease, chronic kidney disease), and length of hospitalization (days). During hospitalization, the following variables were collected: use of proton pump inhibitors, chemotherapy, and antibiotics; diarrhea; intensive care unit admission; and 90-day post-admission mortality rate. In the six months prior to admission, variables analyzed included previous hospitalizations, non-hospital healthcare exposure, and CDI. Additionally, medications administered within two months prior to admission were recorded (antibiotics, glucocorticoids, immunosuppressants, and chemotherapy). Following inclusion in the study, weekly screening for colonization was performed during hospitalization. CDI was defined according to ECDC and ESCMID criteria, requiring diarrhea (≥3 unformed stools within 24 h) together with a positive two-step diagnostic algorithm (GDH antigen screening using the *C. difficile* GDH assay [Certest Biotec, Zaragoza, Spain], followed by confirmatory NAAT using the Xpert *C. difficile* assay [Cepheid, Sunnyvale, CA, USA]) for toxigenic *C. difficile*. Colonization episodes were classified according to a framework adapted from the ECDC criteria for CDI case origin [53], based on hospitalization or non-hospital healthcare contact in the 6 months preceding admission. Episodes were classified as community-associated (CA) if detected within 72 h of admission with no such contact, or as community-onset, healthcare-facility-associated (CO-HCFA) if detected within 72 h but with prior healthcare contact. Episodes detected beyond 72 h were classified as healthcare-facility-onset (HCFO), further stratified as early (72 h–14 days) or late (≥14 days), following the classification proposed by McFarland et al. for nosocomial *C. difficile* acquisition [27].

### 4.2. C. difficile Culture and Antibiotic Susceptibility Testing

A total of 500 µg of stool sample was immersed in 1 mL of 70% ethyl alcohol for 20 min at room temperature. This ethanol shock step selects for the spore-forming fraction of the sample, as *C. difficile* spores are resistant to ethanol exposure, whereas vegetative forms of competing gut microbiota are effectively eliminated [54]. The sample was then centrifuged for 10 min, and the sediment was inoculated onto ChromID *C. difficile* Agar (bioMérieux, Marcy-l’Étoile, France). Plates were subsequently incubated at 36 ± 1 °C for 48 h under strict anaerobic conditions using anaerobic jars and AnaeroGen™ gas-generating sachets (Thermo Scientific, Waltham, MA, USA). The stability of the anaerobic atmosphere was continuously monitored using Oxoid™ Resazurin Anaerobic Indicators (Thermo Scientific) to verify that strictly anaerobic conditions were maintained throughout the incubation period. Presumptive *C. difficile* colonies were identified as grey to black, flat to slightly raised, non-hemolytic colonies with smooth or irregular margins and a matte, non-mucoid appearance, typically 2–5 mm in diameter, resulting from hydrolysis of the chromogenic substrate and its reaction with ferric citrate, allowing for presumptive identification of *C. difficile* from stool samples. Identification of *C. difficile* was performed using the VITEK^®^ MS PRIME (bioMérieux, Marcy-l’Étoile, France) system, which is based on Matrix-Assisted Laser Desorption/Ionization–Time of Flight (MALDI-TOF) technology, for definitive identification. Metronidazole, vancomycin, and moxifloxacin susceptibilities were determined using Etest^®^ strips (bioMérieux, Marcy-l’Étoile, France). The bacterial suspension was adjusted to a 1.0 McFarland turbidity standard, in accordance with the manufacturer’s instructions and the Clinical and Laboratory Standards Institute (CLSI) M11 reference standard for anaerobic bacteria [55].

Minimum inhibitory concentrations (MICs) were read at the point of intersection between the zone of complete inhibition and the MIC scale. Vancomycin and metronidazole MICs were interpreted according to the EUCAST Clinical Breakpoint Tables, version 16.1 (2026). For moxifloxacin, the epidemiological cut-off value (ECOFF) available in the EUCAST MIC distribution database was used to distinguish wild-type from non-wild-type isolates (accessed in December 2025) (Table 5).

**Figure 5:**
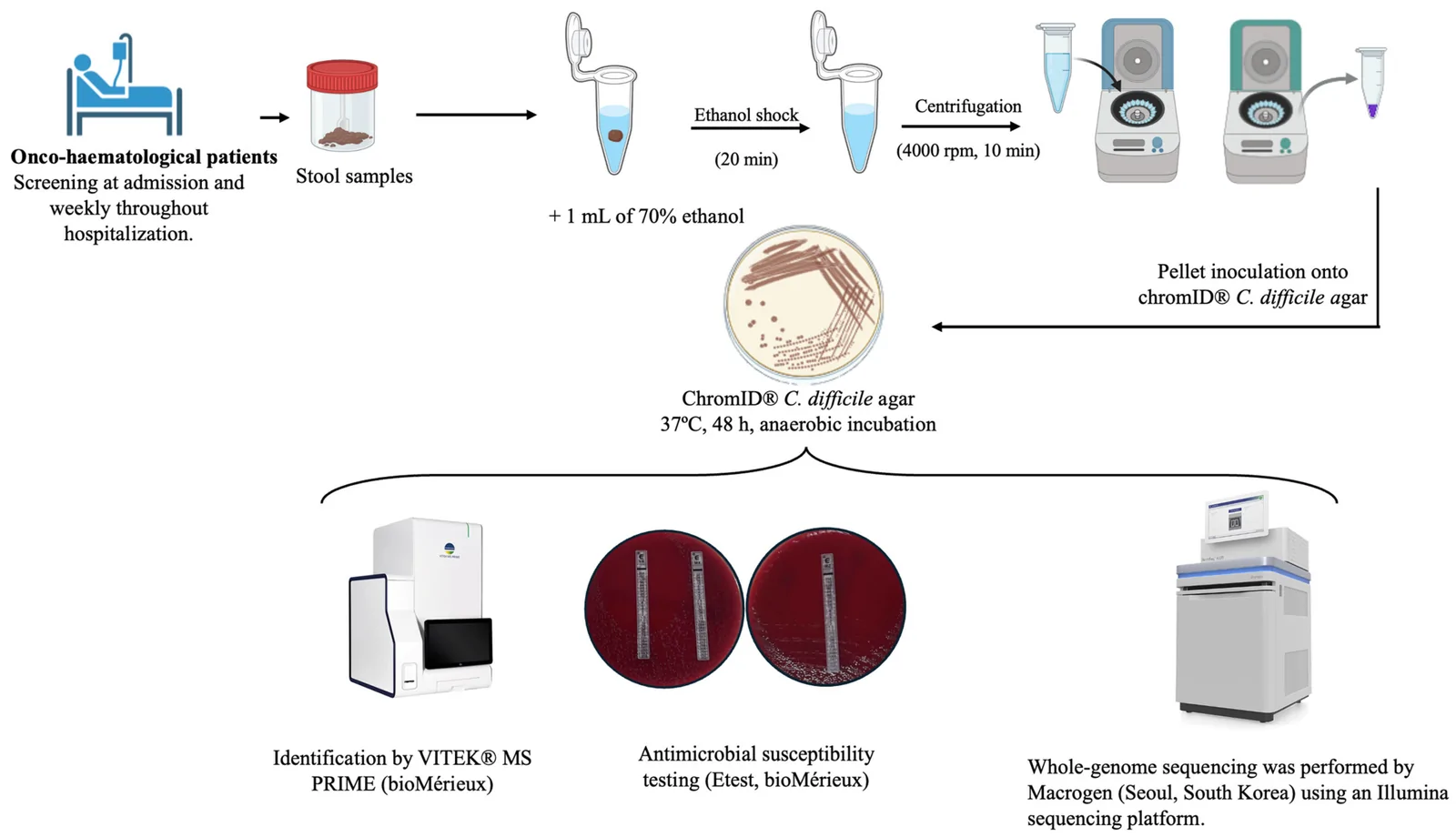
Sample processing summary diagram.

The laboratory workflow, from stool sample processing to isolate identification, antimicrobial susceptibility testing, and whole-genome sequencing, is summarized in Figure 5.

### 4.3. DNA Extraction, Whole-Genome Sequencing and Ribotyping

Each *C. difficile* isolate was cultured in thioglycolate broth under anaerobic conditions for 24 h. The tubes were then centrifuged at 4000 rpm for 20 min. The resulting pellet was transferred into microcentrifuge tubes (Eppendorf, Hamburg, Germany) and centrifuged at 10,000 rpm for 10 min, and the supernatant was discarded. DNA extraction was performed using the QIAamp DNA Mini Kit (Qiagen, Hilden, Germany) according to the manufacturer’s instructions. Genomic DNA was sent to Macrogen Inc. (Seoul, South Korea) for whole-genome sequencing (WGS). Library preparation was performed using Nextera DNA XT Kit according to the manufacturer’s instructions. Sequencing was carried out on an *Illumina system*, generating 151 bp paired-end reads.

The ribotyping of the *C. difficile* isolates was conducted following the ECDC’s Laboratory procedures for diagnosis and typing of human *C. difficile* infection [56,57]. The amplification was performed using the primer set designed by Bidet et al. (FAM-5′-GTGCGGCTGGATCACCTCCT-3′ (16S) and 5′-CCCTGCACCCTTAATAACTTGACC-3′ (23S)). The 16S primers were labeled at the 5′ end with 6-carboxyfluorescein (6-FAM) [54]. Fragment analysis using capillary gel electrophoresis was carried out by the Genomics Service of the University of La Laguna using an AB3500 Analyzer (Applied Biosystems, Foster City, CA, USA), GeneScan 600 LIZ dye Size Standard v2.0, and POP-7 Polymer (Applied Biosystems, Foster City, CA, USA). Ribotype assignment was performed using the Webribo database (https://webribo.ages.at (accessed on 1 April 2025)) [58]. In cases identified as new ribotype, we repeated PCR with primers FAM-5′GCTGGATCACCTCCTTTCTAAG 3′ (Janezicfor) and 5′ TGACCAGTTAAAAAGGTTTGATAGATT 3′ (Janezicrev).

### 4.4. Multilocus Sequence Typing, Acquired Resistance and Virulence Genes

Reads from the sequencer were quality-trimmed using Trimmomatic v0.39 (LEADING:10 TRAILING:15 SLIDINGWINDOW:4:20 MINLEN:100) [59]. Trimmed reads were assembled de novo using SPAdes 4.2.0 with k-mer sizes 33, 55, 77 and 99 in assembly-only mode [60]. Sequence typing was performed on the SPAdes assemblies using the mlst tool v2.33.1 (Torsten Seemann) against the PubMLST *C. difficile* scheme (adk, atpA, dxr, glyA, recA, sodA, tpi) [61]. An ST was assigned only when all seven loci matched an existing profile with 100% identity/coverage; assignments were cross-checked against the PubMLST REST API (downloaded 11 March 2026).

Acquired resistance determinants and chromosomal point mutations were identified with AMRFinderPlus against its curated reference database, with acquired genes additionally cross-validated against ResFinder (v4.7.2) and ABRICATE(v1.4.0) [62,63,64].

The presence of virulence-associated genes was assessed using ABRICATE against the VFDB database together with targeted BLASTN 2.17.0+ searches against full-length reference sequences for a curated set of clinically relevant genes, rather than relying solely on the short fragment references used by VFDB [65,66]. For each isolate, gene presence was called when the summed alignment coverage across all matching contigs reached ≥50% of the reference length at ≥80% nucleotide identity; hits below these thresholds, or those interrupted by a contig boundary, were flagged for manual inspection rather than scored as present or absent.

Detection of non-functional, truncated toxin genes. Toxin genes (*tcdA*, *tcdB*, *cdtA*, *cdtB*) were screened by BLASTN 2.17.0+ against full-length reference sequences, with coverage calculated relative to the complete gene rather than to short database fragments [65]. For alignments covering only part of the reference, the contig’s read coverage was first checked against the genome-wide average to exclude low-coverage assembly artefacts; the genomic region spanning the gap was then extracted and translated in silico from the start codon. A gene was classified as non-functional/truncated only when translation showed a frameshift with a premature stop codon, and this same breakpoint and truncated protein were independently reproduced in more than one isolate—ruling out a single-assembly artefact.

In cases without a truncated binary toxin, detection of truncating mutations in regulatory genes (*cdtR*, *tcdC*) was performed. Isolates flagged by AMRFinderPlus with the “INTERNAL_STOP” category for *cdtR* or *tcdC* were confirmed by extracting the corresponding genomic region and translating it in silico from the start codon, then aligning the resulting protein against the curated NCBI reference (*cdtR*: WZT68956.1; *tcdC*: AJP10340.1) to pinpoint the exact position of the premature stop codon. A mutation was accepted as genuine only when identical in more than one independent isolate.

Phylogenetic tree construction and visualization. Pairwise genomic distances (MinHash/Mash-like k-mer approach, k = 21, sketch size = 2000) were used to build a midpoint-rooted neighbor-joining tree (Biopython), since reference-based SNP calling (Snippy) failed for this dataset; no bootstrap support was computed [67,68]. The tree was visualized in R (ape v5.8.1 [69]) alongside a heatmap of acquired resistance genes (present/absent, or partial when coverage was anomalously low) and non-intrinsic virulence genes (percent identity to the reference).

### 4.5. Data Analysis

Categorical variables were summarized as absolute frequencies and percentages, whereas continuous variables were expressed as median and interquartile range (IQR). Comparisons between groups were performed using Fisher’s exact test for categorical variables and the Mann–Whitney U test for continuous variables. Baseline characteristics were described both at the patient level (n = 215) and at the admission-episode level (n = 262), and analyses were stratified by hospital service (hematology vs. oncology).

To identify factors associated with *C. difficile* colonization, generalized linear mixed models (GLMMs) with a binomial distribution and logit link function were fitted at the admission-episode level. Patient identification was included as a random intercept to account for repeated admission episodes. Candidate predictors were initially evaluated in univariable GLMM analyses (Table S1), and variables showing statistical significance and considered clinically relevant were subsequently included in the multivariable model. Adjusted odds ratios (aORs) and their corresponding 95% confidence intervals (95% CIs) were calculated as measures of association.

Because 90-day mortality is a non-repeatable, patient-level outcome, this specific comparison was performed at the patient level (n = 215), whereas colonization and subsequent CDI, both episode-specific events, were analyzed at the episode level (n = 262). Given that only one confirmed case of CDI occurred during the study period, the association between colonization status and progression to clinical infection was assessed using Fisher’s exact test; the odds ratio was not calculated due to a zero-cell count, precluding a stable point estimate.

All statistical analyses were performed using R version 4.6.1 (R Foundation for Statistical Computing, Vienna, Austria). A two-sided *p*-value < 0.05 was considered statistically significant.

### 4.6. Declaration of Generative AI and AI-Assisted Technologies in the Manuscript Preparation Process

During the preparation of this work, the authors used Claude 3.5 Sonnet (Anthropic) to assist in writing and structuring Python (v3.14.5), R (v. 4.6.1), and Bash (v5.3) scripts for bioinformatic data processing (including pipeline execution, BLASTN alignments, coverage/identity calculations, and in silico translation), as well as drafting portions of Section 4 (multilocus sequence typing, acquired resistance, and virulence genes). All AI-generated code, analytical outputs, and manuscript text were thoroughly reviewed, debugged, and verified by the authors prior to execution and publication, to ensure correctness and reproducibility.

## 5. Conclusions

The progression to active CDI in this cohort of onco-hematological patients was exceptionally low, which is consistent with the low conversion rate observed in clinical practice. Nonetheless, continued genomic surveillance remains essential to identify circulating hypervirulent ribotypes and their clinical significance, and to track the emergence and spread of resistance and virulence determinants among both colonizing and infecting *C. difficile* isolates. Furthermore, the identification of healthcare contact as a near-universal feature among colonized patients highlights the need to strengthen environmental hygiene measures in healthcare settings and to reinforce the implementation of antimicrobial stewardship programs.

## Figures and Tables

**Table 1:** Clinical and demographic characteristics for admitted patients.

Characteristic	Hematology N = 118	Oncology N = 97	*p*-Value	Total N = 215
Men, n (%)	60 (50.8%)	46 (47.4%)	0.681	106 (49.3%)
Age (years), median (IQR)	66 (60–76)	66 (54–73)	0.171	66 (58–75)
Comorbidities, n (%)				
DM2	38 (32.2%)	29 (29.9%)	0.768	67 (31.2%)
Cardiovascular diseases	68 (57.6%)	55 (56.7%)	1.000	123 (57.2%)
Obesity	16 (13.6%)	10 (10.3%)	0.532	26 (12.1%)
Hypothyroidism	14 (11.9%)	6 (6.2%)	0.167	20 (9.3%)
Dyslipidemia	42 (35.6%)	28 (28.9%)	0.310	70 (32.6%)
Chronic obstructive pulmonary disease	8 (6.8%)	2 (2.1%)	0.118	10 (4.7%)
Chronic kidney disease	8 (6.8%)	4 (4.1%)	0.553	12 (5.6%)
Cancer type, n (%)			<0.001	
Non-Hodgkin lymphoma	34 (28.8%)	7 (7.2%)		41 (19.1%)
Plasma cell neoplasm	30 (25.4%)	0 (0.0%)		30 (14.0%)
Acute leukemia	29 (24.6%)	0 (0.0%)		29 (13.5%)
Lymphoid hematologic neoplasms	4 (3.4%)	0 (0.0%)		4 (1.9%)
Myeloid hematologic neoplasms/leukemias	8 (6.8%)	0 (0.0%)		8 (3.7%)
Non-malignant hematologic disease	11 (9.3%)	0 (0.0%)		11 (5.1%)
Lung solid tumor	0 (0.0%)	19 (19.6%)		19 (8.8%)
Gastrointestinal solid tumor ^1^	0 (0.0%)	20 (20.6%)		20 (9.3%)
Breast solid tumor	0 (0.0%)	13 (13.4%)		13 (6.0%)
Pancreatic and biliary tract solid tumor	0 (0.0%)	12 (12.4%)		12 (5.6%)
Other solid tumors ^2^	1 (0.8%)	26 (26.8%)		27 (12.6%)

^1^ Includes hepatic tumors. ^2^ Includes urological, gynecological, central nervous system, and mesenchymal (sarcomas and related) solid tumors. Table 2 presents bivariate comparisons stratified by hospital service for descriptive purposes; variable selection for the multivariable model used a separate, non-stratified univariable screening of all 262 episodes (Table S1).

**Table 2:** Risks factors for developing *C. difficile* colonization stratified by hospital service (episode level, n = 262).

Characteristic	Hematology			Oncology		
	CD Colonized N = 11	CD Not Colonized N = 141	*p*-Value	CD Colonized N = 12	CD Not Colonized N = 98	*p*-Value
Men, n (%)	7 (63.6%)	73 (51.8%)	0.540	5 (41.7%)	50 (51.0%)	0.761
Age (years), median (IQR)	65 (40–75.5)	66 (60–75)	0.522	65 (54.8–68)	67 (54–74)	0.434
DM2	3 (27.3%)	44 (31.2%)	1.000	5 (41.7%)	29 (29.6%)	0.509
Cardiovascular diseases	4 (36.4%)	84 (59.6%)	0.204	7 (58.3%)	57 (58.2%)	1.000
Obesity	1 (9.1%)	20 (14.2%)	1.000	2 (16.7%)	8 (8.2%)	0.299
Hypothyroidism	2 (18.2%)	19 (13.5%)	0.650	1 (8.3%)	8 (8.2%)	1.000
Dyslipidemia	2 (18.2%)	47 (33.3%)	0.504	4 (33.3%)	26 (26.5%)	0.732
Chronic obstructive pulmonary disease	0 (0.0%)	10 (7.1%)	1.000	0 (0.0%)	2 (2.0%)	1.000
Chronic kidney disease	0 (0.0%)	12 (8.5%)	0.602	2 (16.7%)	4 (4.1%)	0.128
Length of hospitalization (days), median (IQR)	19 (8–22.5)	16 (8–29)	0.768	15 (7–33.8)	10 (6–18)	0.364
During hospitalization						
Proton pump inhibitors (PPIs)	11 (100.0%)	135 (95.7%)	1.000	11 (91.7%)	93 (94.9%)	0.509
Chemotherapy	8 (72.7%)	76 (53.9%)	0.347	7 (58.3%)	17 (17.3%)	0.004
Antibiotics	10 (90.9%)	105 (74.5%)	0.297	9 (75.0%)	63 (64.3%)	0.539
Six months prior to admission						
Previous hospitalization	7 (63.6%)	77 (54.6%)	0.755	7 (58.3%)	56 (57.1%)	1.000
Non-hospital healthcare exposure	9 (81.8%)	126 (89.4%)	0.355	9 (75.0%)	93 (94.9%)	0.041
CDI (previous episode)	1 (9.1%)	3 (2.1%)	0.262	0 (0.0%)	0 (0.0%)	1.000
Medications within 2 months prior to admission						
Antibiotics	5 (45.5%)	59 (41.8%)	1.000	7 (58.3%)	33 (33.7%)	0.117
Glucocorticoids or immunosuppressant	6 (54.5%)	96 (68.1%)	0.506	9 (75.0%)	72 (73.5%)	1.000
Chemotherapy	5 (45.5%)	70 (49.6%)	1.000	7 (58.3%)	52 (53.1%)	0.769

Categorical variables: n (%), Fisher’s exact test. Continuous variables: median (IQR), Wilcoxon rank-sum test.

**Table 3:** Adjusted risk factors for *C. difficile* colonization (generalized linear mixed model).

Variable	aOR	95% CI	*p*-Value
Hospital service (hematology vs. oncology, reference)	0.37	0.14–0.96	0.042
Chemotherapy administration during hospitalization	4.16	1.56–11.10	0.004

aOR: adjusted odds ratio; CI: confidence interval. Model: Colonization ~ Service + Chemotherapy (during hospitalization) + (1|Patient ID). Twenty candidate predictors were screened individually prior to model selection (Table S1); given the limited number of colonization events (23 of 262 episodes), the multivariable model was restricted to two predictors.

**Table 4:** Distribution of MLST, ribotype, toxin gene profiles, and regulatory mutations among *C. difficile* strains.

Strain	MLST	Ribotype	*tcdA*	*tcdB*	*cdtA*	*cdtB*	CDT Locus Status	*cdtR*	*cdtR* Mutation	*tcdC*	*tcdC* Mutation	gyrA_T82I
11B	ST458	–	Neg	Neg	Neg	Neg	Absent	Neg	–	Neg	–	Not detected
32	ST11	–	Pos	Pos	Pos	Pos	Intact (full-length)	Pos	Nonsense mutation (codon 322)	Pos	*tcdC-A* genotype (non-functional)	Present
44A	ST15	RT010	Neg	Neg	Neg	Neg	Absent	Neg	–	Neg	–	Not detected
47A	ST2	RT020	Pos	Pos	Pos	Pos	Truncated (~1.9 kb deletion) †	Pos	No mutation reported	Pos	No mutation reported	Not detected
73C	ST238	–	Neg	Neg	Neg	Neg	Absent	Neg	–	Neg	–	Not detected
85	ST15	RT010	Neg	Neg	Neg	Neg	Absent	Neg	–	Neg	–	Not detected
92	ST42	RT106 (probable) ‡	Pos	Pos	Pos	Pos	Truncated (~1.9 kb deletion) †	Pos	No mutation reported	Pos	No mutation reported	Present
95	ST2	RT451	Pos	Pos	Pos	Pos	Truncated (~1.9 kb deletion) †	Pos	No mutation reported	Pos	No mutation reported	Not detected
120	ST26	–	Neg	Neg	Neg	Neg	Absent	Neg	–	Neg	–	Present
128	ST42	RT106	Pos	Pos	Pos	Pos	Truncated (~1.9 kb deletion) †	Pos	No mutation reported	Pos	No mutation reported	Present
132	ST42	RT106 (probable) ‡	Pos	Pos	Pos	Pos	Truncated (~1.9 kb deletion) †	Pos	No mutation reported	Pos	No mutation reported	Not detected
138	ST11	RT078	Pos	Pos	Pos	Pos	Intact (full-length)	Pos	Nonsense mutation (codon 322)	Pos	*tcdC-A* genotype (non-functional)	Present
195	ST55	–	Pos	Pos	Pos	Pos	Truncated (~1.9 kb deletion) †	Pos	No mutation reported	Pos	No mutation reported	Not detected
200	ST2	RT020	Pos	Pos	Pos	Pos	Truncated (~1.9 kb deletion) †	Pos	No mutation reported	Pos	No mutation reported	Not detected
206	ST12	RT003	Pos	Pos	Pos	Pos	Truncated (~1.9 kb deletion) †	Pos	No mutation reported	Pos	No mutation reported	Not detected
224	ST238	–	Neg	Neg	Neg	Neg	Absent	Neg	–	Neg	–	Not detected

† Truncated: ~1.9 kb structural deletion spanning the 3′ end of *cdtA* and 5′ end of *cdtB*, rendering the binary toxin locus non-functional despite gene detection. ‡ Ribotype inferred from MLST sequence type based on previously published ST–ribotype correlations (not confirmed by PCR); interpret as likely, not confirmed [16,17,18].

**Table 5:** Distribution MLSTs and MICs of vancomycin, metronidazole and moxifloxacin of diferent *C. difficile* strains.

Strain	ST	MIC (mg/L) Vancomycin	MIC (mg/L) Metronidazole	MIC (mg/L) Moxifloxacin	Quinolone-Resistance Genes
		ECOFF 2 mg/L	ECOFF 2 mg/L	ECOFF 4 mg/L	
11B	ST458	0.125	0.032	0.125	*gyrB_S366A*
32	ST11	0.25	0.125	>32	*gyrA_T82I* + *gyrB_S366V* + *gyrB_S416A*
44A	ST15	0.125	0.016	0.5	Negative
47A	ST2	0.064	0.064	0.5	Negative
73C	ST238	0.064	0.016	0.25	*gyrB_S366A*
85	ST15	0.125	0.032	0.125	*Negative*
92	ST42	0.064	0.016	>32	*gyrA_T82I*
95	ST2	0.064	0.016	0.25	Negative
120	ST26	-	-	-	*gyrA_T82I*
128	ST42	0.125	0.064	>32	*gyrA_T82I*
132	ST42	0.125	0.016	0.016	Negative
138	ST11	-	-	-	*gyrA_T82I* + *gyrB_S366V* + *gyrB_S416A*
195	ST55	-	-	-	Negative
200	ST2	-	-	-	Negative
206	ST12	-	-	-	Negative
224	ST238	0.125	0.016	12	*gyrB_S366A*

## Data Availability

The whole-genome sequencing data generated in this study are openly available in the NCBI BioProject repository under accession number PRJNA1514183 (https://www.ncbi.nlm.nih.gov/bioproject/PRJNA1514183) (accessed on 10 September 2026). The clinical data supporting the findings of this study are available from the corresponding author upon reasonable request. These data are not publicly available due to privacy and ethical restrictions related to patient confidentiality.

## Supplementary Materials

The following supporting information can be downloaded at: https://doi.org/10.5281/zenodo.21970607 (accessed on 14 September 2026). Table S1: Univariable screening of candidate risk factors for *C. difficile* colonization.

## Abbreviations

The following abbreviations are used in this manuscript:

CDI	*Clostridioides difficile* infection
NTCD	Non-toxigenic *Clostridioides difficile*
MLST	Multilocus sequence typing
ST	Sequence type
RT	Ribotype
MIC	Minimum inhibitory concentration
ECOFF	Epidemiological cut-off value
aOR	Adjusted odds ratio
CI	Confidence interval
GLMM	Generalized linear mixed model
PPI	Proton pump inhibitor
CA	Community-associated
CO-HCFA	Community-onset, healthcare-facility-associated
HCFO	Healthcare-facility-onset
IQR	Interquartile range
ECDC	European Centre for Disease Prevention and Control
